# Genetic polymorphisms in glutathione S-transferase (GST) superfamily and risk of arsenic-induced urothelial carcinoma in residents of southwestern Taiwan

**DOI:** 10.1186/1423-0127-18-51

**Published:** 2011-07-29

**Authors:** Ling-I Hsu, Wu-Ping Chen, Tse-Yen Yang, Yu-Hsin Chen, Wann-Cheng Lo, Yuan-Hung Wang, Ya-Tang Liao, Yu-Mei Hsueh, Hung-Yi Chiou, Meei-Maan Wu, Chien-Jen Chen

**Affiliations:** 1 Genomics Research Center, Academia Sinica, No.128 Academia road, Sec 2, Nankang, Taipei 115, Taiwan; 2 School of Public Health, Taipei Medical University, 250 Wu-Xin Street, Taipei 110 Taiwan; 3 Department of Urology, Taipei Medical University Hospital, 250 Wu-Xin Street, Taipei 110; 4 Division of Environmental Health and Occupational Medicine, National Health Research Institutes, Taiwan

## Abstract

**Background:**

Arsenic exposure is an important public health issue worldwide. Dose-response relationship between arsenic exposure and risk of urothelial carcinoma (UC) is consistently observed. Inorganic arsenic is methylated to form the metabolites monomethylarsonic acid and dimethylarsinic acid while ingested. Variations in capacity of xenobiotic detoxification and arsenic methylation might explain individual variation in susceptibility to arsenic-induced cancers.

**Methods:**

To estimate individual susceptibility to arsenic-induced UC, 764 DNA specimens from our long-term follow-up cohort in Southwestern Taiwan were used and the genetic polymorphisms in GSTM1, GSTT1, GSTP1 and arsenic methylation enzymes including GSTO1 and GSTO2 were genotyped.

**Results:**

The GSTT1 null was marginally associated with increased urothelial carcinoma (UC) risk (HR, 1.91, 95% CI, 1.00-3.65), while the association was not observed for other GSTs. Among the subjects with cumulative arsenic exposure (CAE) ≥ 20 mg/L*year, the GSTT1 null genotype conferred a significantly increased cancer risk (RR, 3.25, 95% CI, 1.20-8.80). The gene-environment interaction between the GSTT1 and high arsenic exposure with respect to cancer risk was statistically significant (multiplicative model, *p *= 0.0151) and etiologic fraction was as high as 0.86 (95% CI, 0.51-1.22). The genetic effects of GSTO1/GSTO2 were largely confined to high arsenic level (CAE ≥ 20). Diplotype analysis showed that among subjects exposed to high levels of arsenic, the AGG/AGG variant of GSTO1 Ala140Asp, GSTO2 5'UTR (-183)A/G, and GSTO2 Asn142Asp was associated with an increased cancer risk (HRs, 4.91, 95% CI, 1.02-23.74) when compared to the all-wildtype reference, respectively.

**Conclusions:**

The GSTs do not play a critical role in arsenic-induced urothelial carcinogenesis. The genetic effects of GSTT1 and GSTO1 on arsenic-induced urothelial carcinogenesis are largely confined to very high exposure level.

## Background

Arsenic (As) exposure is an important public health issue worldwide and more than 100 million people are exposed to arsenic-contaminated water supplies that contain arsenic at a level higher than the internationally-accepted standard (10 μg/L in Taiwan and USA). Chronic arsenic ingestion induces adverse health effects in humans, including black-foot disease [1,2], ischemic heart disease [3,4], hypertension [5], diabetes mellitus [6], cerebrovascular and microvascular diseases [7,8] and various cancers [9,10]. A strong association of arsenic exposure with an increased incidence of bladder cancer has been observed in the southwest (high-exposure area) and northeast (moderate-exposure area) regions of Taiwan [11,12]. Even with a relatively low exposure level, the association of arsenic with bladder cancer risk has also been observed in Finnish study [13]. Nonetheless, among relatively homogeneously exposed people, the disease manifestations are diverse, which suggests that there is a marked variation in susceptibility among individuals. Nutritional status, ethnicity, an early age of exposure and variations in arsenic biotransformation are all potentially responsible for differences in individual susceptibility to arsenic-induced carcinogenesis.

Most mammals, including humans, metabolize inorganic arsenic via arsenic methylation to a range of different metabolites that have different toxic potencies [14,15]. The classic methylation pathway involves reduction and methylation reactions via one-carbon metabolism. Pentavalent arsenicals such as arsenate As(V) or monomethylarsonic acid [MMA(V)] can be reduced to trivalent arsenite [As(III)] or monomethylarsinic acid [MMA(III)] respectively; and then methyl groups from S-adeno methionine (SAM) are used for further methylation to form the metabolites monomethylarsonic acid [MMA(V)] and dimethylarsinic acid [DMA(V)]. Variation in the production and excretion of these arsenic metabolites could explain individual variation in arsenic toxicity [16]. Recent studies have revealed that arsenic metabolic capacity may be an important risk-modifying factor for arsenic-induced health effects. Inefficient methylation of arsenic has been documented to be a significant modifier for arsenic-induced skin lesions and cancers, bladder cancer, peripheral arterial disease, hypertension and carotid atherosclerosis [17-21].

The efficiency of arsenic methylation can be influenced by genetic polymorphisms within individuals [22,23]. Glutathione S-transferase omega (GSTO) and arsenic (III) methyltransferase (AS3MT, or CYT19) are involved in classic arsenic methylation in a variety of animals including humans [24-26]. The GSTOs, including GSTO1 and GSTO2, can catalyze the reduction of MMA(V) to MMA(III), which is thought to be the rate-limiting step of arsenic methylation in humans [27]. The relationship between the GSTOs and arsenic metabolism has been explored to test if GSTO polymorphisms can explain variation in arsenic methylation capacity as well as variation in individual susceptibility to arsenic exposure. Most studies have not shown a significant association of this gene with extreme urinary profiles [28-30]. However, a recent study in Taiwan showed that the GSTO2 Asn142Asp (N142D) homo-variant was associated with an increased iAs% [31]. A study of a northern Mexican population also suggested that there is an association between GSTO1 E155del and an increased percentage of inorganic arsenic, either As^III ^or As^V ^[32]. The association of GSTOs with As-induced health effects has also been examined in several arseniasis areas. The polymorphisms in GSTOs could be a significant modifier for arsenic-induced skin lesions and urothelial carcinoma (UC) [20,33]. For the purpose to further clarify the effect of GSTOs as well as other GST family members on arsenic-induced urinary cancers, we monitored a cohort of 764 subjects established in Southwest Taiwan in 1988 and single nucleotide polymorphisms (SNPs) genotyping was performed. The association of GSTM1, GSTT1, GSTP1, GSTO1 and GSTO2 with UC risk was examined. In addition, the joint effect of such genes with levels of arsenic exposure on cancer risk was also examined.

## Materials and methods

### Study cohort

Putai township, located on the southwestern coast of Taiwan, is a township where blackfoot disease (BFD) is endemic and it has an overall BFD prevalence of about 2.2/1000. The residents in Putai consumed artesian well water (100-300 meters deep) for more than 50 years. Three villages, Homei, Fuhsin, and Hsinming in Putai Township have the highest BFD prevalences at 13.6, 9.6, and 10.3/1000, respectively [34]. These villages were selected as the study area. The median arsenic concentrations of the artesian well water ranged from 0.70 ppm to 0.93 ppm in the study area. The residents stopped artesian water consumption in the 1970s when a tap water supply system was implemented. From a total of 2258 residents aged 30 or older who were registered in the study area, only 1571 who lived at least 5 days a week in the village were recruited into the study in 1988. Twenty-five subjects were excluded from the study either due to previous cancer history or incomplete personal identification information, and finally a total of 1546 subjects were recruited for the study. All the subjects were of the same Ming-Nang ethnicity. Between 1989 and 1996, about 1081 study subjects underwent six community health examinations. At this time, biological specimens were collected including urine, blood buffy coat, hair, and nails. The buffy coat samples were stored at -80°C for DNA extraction at a later date. About 30% subjects refused to give the blood samples or had inadequate DNA samples. There were no differences in characteristics between the subjects with and without blood samples based on age, gender, educational level and smoking status. A total of 764 (71%) adequate lymphocyte DNA samples were available for SNP analysis.

A standardized personal interview based on a structured questionnaire was carried out in 1988 to collect information on risk factors including sociodemographic characteristics, lifetime residential and occupational histories, drinking water supply, cigarette smoking, alcohol drinking, as well as personal and family history of disease. Detailed histories of residency and duration of drinking artesian well water were used to derive a cumulative arsenic exposure. The cumulative arsenic exposure (CAE) of each subject was defined as Σ(C*i *× D*i*), where C*i = *the median arsenic level (mg/L) in well water of the *i*th village the subject had lived and D*i = *the duration (years) of drinking artesian water in the *i*th village [35]. CAE was calculated only for those subjects for whom there was complete information on arsenic exposure due to drinking artesian well water throughout his or her lifetime. The CAE was set at "0" when the subjects consumed water with arsenic concentrations equal or less than 10 μg/L.

### Cancer Incidence

Individuals' unique national identification was used to link with the national cancer registry profile in Taiwan to identify the cancer status of each cohort subject. The cancer registry system was implemented in 1978 and the complete information for cancer was updated regularly for the whole Taiwan population.

### SNP Genotyping

The GSTM1 and GSTT1 primer pairs (table 1) were mixed with β-globulin primer (5'-CAA CTT CAT CCA CGT TCA CC-3' and 5'-GAA GAG CCA AGG ACA GGT AC-3') in a multiplex polymerase chain reaction (PCR). PCR was performed in a total volume of 100 μL, containing 10 μL of genomic DNA (50-100 ng), 400 ng of each of the above primers, and 5 units of Taq polymerase (SuperTag, Protech, Taiwan). The reaction was incubated at 94°C for 4 min and subjected to 35 cycles of 94°C for 60 s, 55°C for 60 s and 72°C for 60 s, then a final 72°C-extension for 10 min. Next, 10-μL PCR aliquots were electrophoresed on 2% agarose gels and were stained with ethidium bromide. The internal standard fragment of α-globulin was 268-bp in length, whereas the amplified gene products of GSTM1 and GSTT1 were 215 bp and 480 bp, respectively.

**Table 1:** The description of SNPs of arsenic-metabolized enzyme and the primers used for realtime PCR

Enzyme	Base change	Amino acid change	Variant allele frequency	Accession number	Primer pairs
GSTM1	Gene deletion	No protein	0.5749		5'-GAA CTC CCT GAA AAG CTA AAG C-3' & 5'-GTT GGG CTC AAA TAT ACG GTC G-3'
GSTT1	Gene deletion	No protein	0.4966		5'-TTC CTT ACT GGT CCT CAC ATC TC-3' & 5'-TCA CCG GAT CAT GGC CAG CA-3'
GSTP1	Exon 5 A→G	Ile105Val (I105V)	0.2256	rs1695	5'-ACC CCA GGG CTC TAT GGG AA-3' & 5'-CAG GTT GTA GTC AGC GAA G-3'
GSTO1	Exon 4 C→A	Ala140Asp (A140D)	0.1660	rs4925	C_11309430_30
GSTO1	Exon 4 -/AGG	Glu155del (E155del)	0.0125	rs11509437	Forward: 5'-TCTAGGTGCCATCC TTG-3' Reverse: 5'-TGATAGCTAGGAGAAATAA-3'
GSTO1	Exon 6 C→T	Glu208Lys (K208E)	0.0125	rs11509438	C___11309432_20
GSTO1	Exon6 A→C	Thr217Asn (T217N)	0.0000	rs15032	C___1174185_80
GSTO2	Exon2 5'UTR(-183) A→G	---	0.2032	rs2297235	C___3223142_1_
GSTO2	Exon5 A→G	Asn142Asp (N142D)	0.2467	rs156697	C___3223136_1_

Determination of genotype at the GSTP1 locus by the PCR-restriction fragment length polymorphism (RFLP) method and the primers for PCR are 5'-ACC CCA GGG CTC TAT GGG AA-3' and 5'-CAG GTT GTA GTC AGC GAA G-3'. The reaction was incubated at 94°C for 4 min and subjected to 35 cycles of 94°C for 30 s, 60°C for 30 s, 72°C for 30 s, and a final 72°C-extension for 5 min. The PCR products were digested with 5 U of Alw261 (New England Biolabs, Beverly, MA) and the products were separated on 3% agarose gels. The GSTP1 I105V A/G polymorphisms were classified as homozygous A/A (major allele), heterozygous for A/G, or homozygous for G/G (minor allele).

Real-time PCR: Genotyping for polymorphisms in GSTO1 A140D, K208E, E155del and GSTO2 5'UTR(-183) A/G and N142D was performed using real-time PCR. For each of the SNPs, primer-probe sets were made using the Applied Biosystems design service (Foster City, California). Two fluorogenic minor groove binder probes were designed with different fluorescent dyes to allow single-tube genotyping. The primers for SNP genotyping are listed in table 1. Real-time PCR was performed using 2.5 μL of TaqMan 2× universal master mix (Applied Biosystems, Foster City, CA), 0.05 μL of 40× primer-probe, 0.45 μL of RNase- and DNase-free water, and 2 μL of sample DNA, in a total volume of 5 μL per single tube reaction. DNase-free water served as the nontemplate control and DNA of known genotype was used as a positive control; both were included in each assay run. Assay conditions were 2 min at 50°C, 10 min at 95°C, and 40 cycles of PCR at 95°C for 15 s and 60°C for 1 min. Analysis was performed using the SDS, version 2.0, software. Each sample was verified visually by examining the PCR curves generated to eliminate false positives aberrant light emission.

RFLP for GSTO1 and GSTO2: The GSTO1 A140D genotype was determined by PCR-RFLP. The primers for PCR were 5'-AAA GTT GTT TCT TAA ACG TGC C-3' and 5'-AAG TGA CTT GGA AAG TGG GAA-3'. The reaction was incubated at 95°C for 15 min and subjected to 35 cycles of 94°C for 60 s, 55°C for 60 s, 72°C for 60 s, and a final extension for 10 min at 72°C. The PCR products were digested with Cac8 I (New England Biolabs), and the products were separated on 3% agarose gels. The genotypes were determined as follows: homozygous wild type C/C: 243, 145, and 67 bp; heterozygous C/A: 388, 243, 145, and 67 bp; homozygous variants A/A: 388 and 67 bp.

Determination of GSTO2 N142D genotype by PCR-RFLP: The primers for PCR were 5'-ACT GAG AAC CGG AAC CAC AG-3' and 5'-GTA CCT CTT CCA GGT TG-3'. The reaction was incubated at 95°C for 10 min and subjected to 35 cycles of 95°C for 60 s, 62°C for 60 s, and 72°C for 60 s, followed by a final extension at 72°C for 10 min. The PCR product was digested with MboI (New England Biolabs), and the products were separated on 3% agarose gels. The genotypes were determined as followed: homozygous wild type A/A: 280 bp; heterozygous A/G: 280, 231, and 49 bp; homozygous variants G/G: 231 and 49 bp.

For the GSTO1 A140D and GSTO2 N142D polymorphisms, all the cancer specimens and 20% of the non-cancerous specimens were repeated by RFLP-PCR. The κ statistics were about 0.88 and the samples with discordant results were sent for DNA sequencing for genotype validation. For GSTM1 and T1, all of the cancer samples and 20% of the non-cancer samples were repeated; the samples with discordant results were repeated until two same data were shown for the genotypes. For other SNPs, 15% of the samples were run in duplicate and all of the κ statistics were > 0.94. All the samples were relabeled for the experiments and the researchers were blinded to individual identities and results.

### Statistical analysis

The outcome of primary interest was newly-diagnosed UC (formerly urinary transitional cell carcinoma) during the follow up. Individual follow-up person-years were calculated from the entry date into the study to the date of cancer diagnosis, death, or study end on Dec 31, 2007, whichever came first. Among 764 study subjects, the incidence of the disease outcome was calculated and the age-sex adjusted hazard ratio (HR) was estimated using Cox's proportional hazard model according to putative risk factors including age at recruitment (< 49, 50-59, 60+years), sex, education (illiteracy, elementary school, high school or more), cigarette smoking (yes or no), alcohol drinking (yes or no) and CAE (0-9.9, 10.0-19.9, 20+ mg/L*year).

Tests for Hardy-Weinberg equilibrium of each genetic marker among non-cancer subjects were conducted on observed and expected genotype frequencies using Pearson's χ^2 ^test with one degree of freedom. The SNPs with variant frequencies greater than 10% were included for further analysis. The incidence of UC was calculated by the genotypes of selected markers. For GSTM1 and GSTT1, genotypes were dichotomized into two categories (null and non-null genotypes). For GSTP1, GSTO1, GSTO2, genotypes were categorized into three groups (major allele homozygous, heterozygous, and homozygous variant). Cox proportional hazard regression analyses were used to estimate HRs and 95% confidence intervals (CIs) for associations between genotypes of interest and outcome, controlling for the following putative risk factors including age (continuous), gender, educational level, and cigarette smoking. Linkage disequilibrium was analyzed by calculating D' values for GSTO1 A140D, GSTO2 5'UTR(-183)A/G, and GSTO2 N142D. D' is a coefficient of linkage disequilibrium and can be estimated as (p_AB_p_ab _- p_Ab_p_aB_) where p_AB _is a fraction of gamete AB. |D'| values ranged from 1.0 when two polymorphisms were maximally associated and zero when they were randomly associated.

A stratified analysis by arsenic exposure was performed to examine whether the association of the selected markers with arsenic-induced UC depended on exposure level. To maximize the differences between two stratified groups and to avoid "zero" UC case among the subjects with relatively low exposure level and with heterozygous or homozygous variant genotype, the sensitive analysis with various CAE cutoff points was performed to monitor the optimal cutoff point for stratified analyses. We conducted gene-exposure interaction analyses using a regression model; the multivariable-adjusted HR was estimated for each group using subjects with the wild homozygote and low arsenic exposure as a reference. For the non-null and null genotypes, an interaction term was created and a *p *value was estimated by comparing the two models with or without the interaction term. For three genotypes, two dummy variables were created as interaction terms (heterozygous*arsenic group, homozygous variant*arsenic group), and a *p *value was estimated by comparing two models with or without the two interaction variables. The etiologic fraction for the effects of the interaction, an indication of the "departure from additivity ([RR_11_-RR_01_-RR_10_+RR_00_]/RR_11_), was calculated and the 95% CI was estimated using the formula described by Walker [36]. If the case number in the subgroup was zero, we used 0.1 instead to calculate EF and 95%CI. All analyses were performed with SAS statistical software (version 9.1.2, SAS Institute Inc., Cary, NC, USA). The study was approved by the ethics board of the institution prior to starting the study and the informed consent was obtained from all subjects.

## Results

A total of 13,317 person-years were observed during the 18 years of follow-up, with a median period: 16.4 years. By the end of 2007, a total of 41 newly-diagnosed UC had occurred during the follow-up period, yielding an incidence of 307.9 cancers per 100,000 person-years. Table 2 shows the events and follow-up person-years for UC by age at recruitment, sex, educational level, cigarette smoking, alcohol drinking and CAE. Increased UC incidence was found to be associated with older people, being male and having low education level. Education level was inversely associated with the risk of UC in a dose-response manner. Cigarette smoking and alcohol drinking was not significantly associated with cancer risk. The incidence rates of UC per 100,000 person-years was 26.4, 207.2 and 835.1 for CAE < 10.0, 10.0 ≦ CAE < 20.0 and CAE ≥ 20.0, respectively. When compared to the CAE < 10.0 group as the reference, the HRs were 5.96 (95%CI, 0.72-49.03) and 19.31 (95%CI, 2.46-151.24) for the low to high exposure levels with a *p *value 0.0003 for test for trend.

**Table 2:** Univariate analysis of urothelial carcinoma (UC) risk among 764 cohort subjects by age at recruitment, sex, education level, cigarette smoking, alcohol drinking and cumulative arsenic exposure

Risk factors	Count(%)	Follow-up person-years	UC	Crude incidence (per 10^5^)	Age- Sex adjusted HR (95%CI)
**Age at recruitment^a^**					
**< 50**	**390(51.1)**	**7251**	**11**	**151.7**	**ref**
**50-59**	**270(35.3)**	**4508**	**22**	**488.0**	3.38(1.64-6.98)**
**60+**	**104(13.6)**	**1557**	**8**	**513.8**	3.68(1.48-9.18)**
**Sex^b^**					
**Male**	**336(44.0)**	**5716**	**20**	**349.9**	**ref**
**Female**	**428(56.0)**	**7601**	**21**	**276.3**	**0.77(0.42-1.43)**
**Education level**					
**Illiteracy**	**256(33.5)**	**4270**	**22**	**515.2**	**ref**
**Elementary**	**363(47.5)**	**6386**	**18**	**281.9**	**0.64(0.32-1.25)**
**High school and above**	**145(19.0)**	**2662**	**1**	**37.6**	0.11(0.01-0.83)* P_trend_: 0.01
**Cigarette smoking**					
**No**	**595(77.9)**	**10535**	**33**	**313.2**	**ref**
**Yes**	**169(22.1)**	**2781**	**8**	**287.7**	**0.67(0.27-1.64)**
**Alcohol drinking**					
**No**	**660(86.4)**	**11595**	**33**	**284.6**	**ref**
**Yes**	**104(13.6)**	**1721**	**8**	**464.8**	**1.40(0.58-3.35)**
Cumulative arsenic exposure (mg/L*yr)					
**0-9.9**	**206(26.9)**	**3792**	**1**	**26.4**	**ref**
**10.0-19.9**	**188(24.6)**	**3379**	**7**	**207.2**	**5.96 (0.72-49.03)**
**20.0+**	**183(23.9)**	**2874**	**24**	**835.1**	19.31 (2.46-151.24))**
**Unknown**	**187(24.6)**	**3271**	**9**	**275.1**	**7.11 (0.86-58.83)** **P_trend_:0.0003**

a: Adjusted for sex; b: Adjusted for age in a-year increment

UC: urothelial carcinoma; HR: hazard ratio; CI: confidence interval

* p < 0.05; **p < 0.01; ***p < 0.0001

Linkage disequilibrium analysis showed that the GSTO1 Asp140 allele was strongly linked with the GSTO2 5'UTR(-183) G allele and the GSTO2 Asp142 allele (|D'|, 0.9039 and 0.9038, respectively. *p *< 0.0001 for both SNPs). The GSTO1 Glu155 deletion in exon 4 was strongly linked with the Lys 208 allele in exon 6. The incidences and age-sex-adjusted HRs for the SNPs of interest and the GSTO1 A140D-O2 A(-183)G-O2 N142D diplotype are shown in Table 3. The GSTT1 null genotype was found to be significantly associated with an increased cancer risk (HR, 1.91, 95% CI: 1.00-3.65, *p *= 0.05). The GSTO diplotype AGG/AGG was potentially associated with increased cancer risk, but the association was not statistically significant.

**Table 3:** Univariate analysis of urothelial carcinoma risk by genotypes of GST superfamily

		Urothelial carcinoma		
		
	No (%)	**Case no**.	HR (95% CI)	P value
GSTM1				
Non-null	312 (42.5)	18	ref	
Null	422 (57.5)	23	0.94(0.51-1.74)	0.84
GSTT1				
Non-null	368 (50.3)	14	ref	
Null	363 (49.7)	27	1.91(1.00-3.65)*	0.05
GSTP1-105				
AA	491 (64.8)	25	ref	
AG	192 (25.3)	10	1.07(0.51-2.23)	0.86
GG	75 (9.9)	5	1.71(0.64-4.56)	0.29
AG+GG	267 (35.2)	15	1.21(0.64-2.31)	0.56
GSTO1-140				
CC	521 (70.0)	27	ref	
CA	199 (26.7)	9	0.81(0.38-1.73)	0.59
AA	24 (3.3)	2	2.11(0.50-8.99)	0.31
CA+AA	223 (30.0)	11	0.92(0.45-1.85)	0.80
GSTO2-(-183)				
AA	487 (64.2)	26	ref	
AG	234 (30.9)	11	0.73(0.36-1.48)	0.38
GG	37 (4.9)	4	2.58(0.88-7.52)	0.08
AG+GG	271 (35.8)	15	0.90(0.48-1.71)	0.75
GSTO2-142				
AA	428 (56.6)	20	ref	
AG	283 (37.4)	17	1.14(0.60-2.19)	0.69
GG	45 (6.0)	4	2.62(0.88-7.83)	0.08
AG+GG	328 (43.4)	21	1.28(0.69-2.37)	0.43
GSTO1(140)/O2(-183)/O2(142)				
CAA/CAA	397 (53.8)	18	ref	
CAA/AGG	163 (22.1)	7	0.83(0.34-1.98)	0.66
AGG/AGG	20 (2.7)	2	3.08(0.70-13.54)	0.13
Others	158 (21.4)	11	1.55(0.73-3.28)	0.25

HR: adjusted for age/gender/cigarette smoking/education level

HR: hazard ratio; CI: confidence interval

* p < 0.05; **p < 0.01; ***p < 0.0001

Based on the sensitive analysis with various cutoff points of arsenic exposure level, it was consistently shown that the gene effect of GSTT1 and GSTO1 A140D was largely confined to high cumulative arsenic exposure (Additional file 1). When CAE cutoff point was 20 mg/l*years, the significant association of both GSTT1 and GSTO1 A140D with UC risk could be observed.

Table 4 shows the stratified analysis of the association between the SNPs and risk of UC according to CAE status with cutoff point as 20 mg/l*years. Significant association of the GSTT1 null genotype with an increased cancer risk was only observed among high exposure group with CAE ≥ 20 (HR, 3.25, 95% CI, 1.20-8.80, *p *= 0.02). The subjects with both a high exposure level and the homozygous variant of GSTO1 Asp140 had an increased HR of 4.79 (95% CI, 1.03-22.39, *p *= 0.05), with incidence rates of 3508.8 per 100,000. Analysis of GSTO1/O2 showed that among the CAE ≥ 20 subjects, the diplotype AGG/AGG had a significantly increased cancer risk compared to CAA/CAA subjects with an estimated HR of 4.91 (95% CI, 1.02-23.74, *p *= 0.05). No increased cancer risk for the AGG/AGG genotype was observed among the subjects with CAE < 20.

**Table 4:** Stratified analysis of urothelial carcinoma risk by genotypes of GSTM1, T1, P1, O1 and O2 according to cumulative arsenic exposure

	CAE < 20 mg/L*yr				CAE ≧ 20 mg/L*yr			
		
	No	**Cs No**.	HR (95% CI)	P value	No	**Cs No**.	HR (95% CI)	P value
GSTM1								
Non-null	165	4	ref		70	10	ref	
Null	211	4	0.69(0.17-2.81)	0.61	110	14	0.90(0.40-2.03)	0.79
GSTT1								
Non-null	194	6	ref		76	5	ref	
Null	180	2	0.27(0.05-1.37)	0.11	104	19	3.25(1.20-8.80)*	0.02
GSTP1-105								
AA	262	7	ref		112	12	ref	
AG	93	1	0.41(0.05-3.35)	0.41	47	7	1.41(0.55-3.62)	0.47
GG	36	0	0.00	0.99	22	4	1.76(0.51-5.73)	0.35
AG+GG	129	1	0.33(0.04-2.66)	0.30	69	11	1.52(0.66-3.48)	0.33
GSTO1-140								
CC	264	6	ref		126	16	ref	
CA	106	1	0.32(0.04-2.73)	0.30	47	4	0.70(0.23-2.10)	0.52
AA	17	0	0.00	0.99	4	2	4.79(1.03-22.39)*	0.05
CA+AA	123	1	0.30(0.04-2.49)	0.26	51	6	0.97(0.37-2.50)	0.94
GSTO2-(-183)								
AA	247	6	ref		115	16	ref	
AG	119	1	0.23(0.03-2.02)	0.19	61	6	0.71(0.28-1.84)	0.48
GG	26	1	2.04(0.23-17.88)	0.52	6	2	2.90(0.61-13.66)	0.18
AG+GG	145	2	0.43(0.09-2.21)	0.32	67	8	0.87(0.37-2.05)	0.74
GSTO2-142								
AA	218	4	ref		100	13	ref	
AG	145	4	1.17(0.28-4.80)	0.83	71	8	0.88(0.37-2.14)	0.78
GG	29	0	0.00	0.99	11	3	2.53(0.71-8.99)	0.15
AG+GG	174	4	1.05(0.26-4.30)	0.94	82	11	1.08(0.48-2.41)	0.86
GSTO1(140)/O2(-183)/O2(142)								
CAA/CAA	205	3	ref		94	12	ref	
CAA/AGG	89	0	0.00	0.99	41	4	0.79(0.25-2.49)	0.69
AGG/AGG	15	0	0.00	1.00	4	2	4.91(1.02-23.74)*	0.05
Others	77	4	3.45(0.75-15.78)	0.11	38	4	0.85(0.27-2.65)	0.78

HR adjusted for age/gender/cigarette smoking/education level * p < 0.05; **p < 0.01; ***p < 0.0001

**Figure 1:**
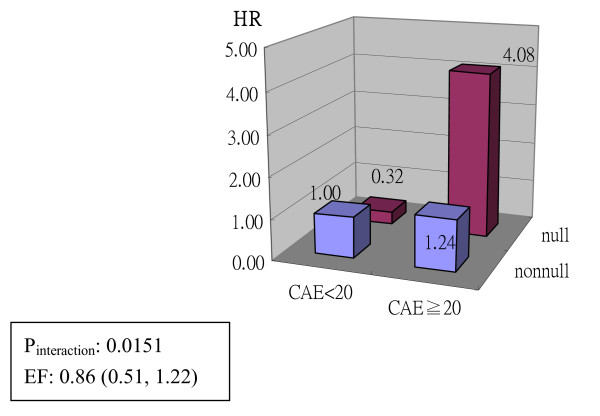
**Interaction analysis between arsenic exposure level and GSTT1 genotype on UC risk**.

**Figure 2:**
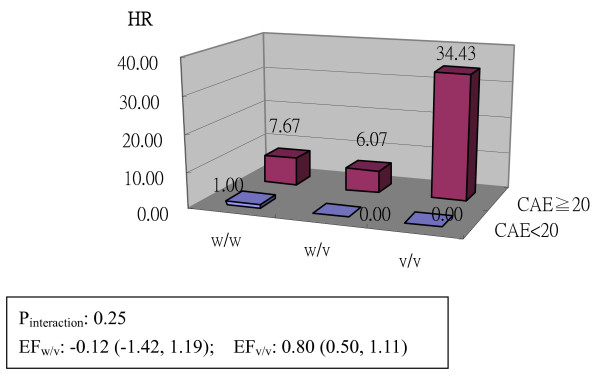
**Interaction analysis between arsenic exposure level and GSTO A140D-O2 5'UTR(-183)A/G-O2 N142D diplotype on UC risk**. ** The case number of subgroup with GSTO w/v or GSTO v/v and low level exposure was assumed as 0.1 to calculate 95% CI of EF_w/v _and EF_v/v_, respectively. HR: adjusted for age/gender/cigarette smoking/education level. Abbreviations HR: hazard ratio; CAE: cumulative arsenic exposure; EF: etiologic fraction; UC: urothelial carcinoma; w/w: wild type homozygous; w/v: heterozygous; v/v: variant homozygous.

GST: glutathione S-transferase; CAE: cumulative arsenic exposure; HR: hazard ratio; CI: confidence interval;

The interaction analysis of GSTT1 and arsenic exposure level on UC risk is shown in Figure 1. The subjects with GSTT1 null genotype and high arsenic exposure had a 4.1-fold higher risk of UC (HR, 4.08, 95% CI, 1.46-11.40, *p *< 0.01) when compared to the subjects with low exposure and the GSTT1 non-null genotype. The interaction was statistically significant in the multiplicative model and the etiologic fraction was 0.86. The interaction analysis of the GSTO1/O2 diplotype and arsenic exposure level on UC risk is shown in Figure 2. The subjects with the GSTO AGG/AGG diplotype and high arsenic exposure had a 34-fold higher cancer risk (HR, 34.43, 95% CI, 5.03-235.74, *p *< 0.01) when compared to the reference and the etiologic fraction was estimated to be 0.80.

## Discussion

Our study was designed to estimate gene effect together with gene and arsenic exposure interaction on the risk of urothelial carcinoma by long-term follow-up study. In the present study, the dose-response relationship between CAE and UC risk was consistent with our previous observations in southwestern and northeastern Taiwan [11,12]. The protective role of education level suggested that a low socioeconomic status was associated with an increased risk of As-induced UC. Cigarette smoking was not significantly associated with UC, suggesting that tobacco use plays a relatively minor role in urinary carcinogenesis in an area that has high exposure to arsenic. We propose the possibility that people who smoke in the arsenic-exposed area have a strong tendency to develop lung cancers because of a significant interaction between smoking and arsenic on lung carcinogenesis [37]. Such a tendency may have the effect of attenuating the association between smoking and UC.

Although urothelial cancer cases were limited in the study, cancer cases among 363 subjects with GSTT1 null was twice as many as the cases among 368 subjects with GSTT1 non-null and a significant association of GSTT1null with UC could be observed with the adjustment of cigarette smoking and potential confounding factors. Moreover, the genetic effect of GSTT1 on cancer risk was largely confined to high arsenic level. The interaction between GSTT1 and high exposure level was statistically significant under the multiplicative model. These observations suggest that GSTT1 may have a significant role in As-induced urothelial carcinogenesis, especially among high exposure level. The current result was inconsistent with the previous studies showing the GSTT1 non-null genotype was associated with an increased risk of As-induced skin lesions [38] and UC [39]. The limited sample size without sufficient power to detect true association may partly explain the discrepancies of these studies. The other possibility for these discrepancies may due to markedly difference of exposure level among these studies. We noticed the protective role of GSTT1 non-null was largely confined to the individuals with cumulative arsenic exposure higher than 20 mg/L*years in the present study. On the other hand, the association of GSTT1 non-null with increased bladder cancer risk was more confined to individuals with relatively low level exposure (most study subjects have consumed contaminated water with arsenic concentration less than 0.2 mg/L for less than 30 years). These observations reveal the possibility that the effects of GSTT1 polymorphism on susceptibility to arsenic-induced urothelial carcinoma depend on arsenic exposure level. The association between GSTs and UC with regard to various arsenic exposure levels needs to be further clarified.

The roles of GSTT1 in arsenic-induced carcinogenesis remain unclear. The modulating effect of GSTs on arsenic methylation has been explored in several studies. Our previous study on the northeast coast of Taiwan has shown that the GSTT1 null genotype was associated with an elevated percentage of dimethylarsonous acid (DMA) in urine [40]. By contrast, the study in Argentina showed that the GSTT1 null was associated with lower percentage of DMA [28]. Modification effect of the GSTT1 null on %MMA and %DMA was also observed in that study. Although these studies show inconsistent results for the association between GSTs and urinary arsenic metabolite pattern, the findings indicate that the GSTT1is possibly responsible for a part of interindividual variation in arsenic metabolism. From current knowledge the GSTT1 does not catalyze the reactions of arsenic methylation directly. Instead, GSTT1 may involve in arsenic methylation by an indirect way, for example, by depletion the glutathione (GSH) level. GSH is required for arsenic metabolism such as iAs(V) or MMA(V) reduction [36,41]. Depletion of GSH might cause decreased efficiency of arsenic methylation. The GSTT1 null may have a protective role on urothelial carcinogenesis if this enzyme can quickly deplete the GSH.

In addition to the possible roles of GSTs in arsenic biotransformation, GSTs are also phase II metabolic enzymes that are involved in detoxification of the xenobiotics by glutathione conjugation. The evidence generally supports the modulating effect of GSTM1 and GSTT1 polymorphisms on cancers closely-related to chemical exposure, including bladder cancer [42,43]. GSTs may prevent DNA damage from endogenously-formed oxidative stress or environmentally-exposed carcinogens [44-46]. There is considerable evidence that reactive oxygen species (ROS) are involved in the genotoxicity of arsenical compounds [47,48]. Our previous study in Northeastern Taiwan showed that the concentration of reactive oxidants of plasma was positively correlated with arsenic concentration in whole blood among arsenic-exposed people [49]. These phase II enzymes, perhaps GSTT1, possibly play an important role to detoxify arsenic-induced ROS. Up to now the direct reactions of GSTs toward arsenic-induced ROS have yet been reported and the relations between GSTs, arsenic-induced ROS and DNA damage need to be further addressed.

For the overall study subjects without considering arsenic exposure level, GSTO1/O2 was not associated with urothelial carcinoma, suggesting the limited role of GSTOs for urothelial carcinogenesis. However, among high As-exposed subjects with 75% UC cases diagnosed in this subgroup (24 among a total of 32 UC cases with known exposure levels), a significant association between GSTO polymorphism and UC was observed. In this highly-exposed group, strikingly high UC incidence was observed (3500 per 100,000) among people with GSTO1/O2 AGG/AGG diplotype. This observation suggests a possible role of GSTOs in individual susceptibility to UC especially at a high exposure level. Our result also supports the previous studies showing the modification effect of GSTOs on As-induced health effects [20,33]. For examples, GSTO2 A424G and A-183G homovariant was associated with a 1.6-fold and a 2.4-fold UC risk, respectively in one hospital-based case-control study [33]. A study in Bangladesh also showed an joint effect of arsenic exposure with GSTOs on As-induced skin lesions [20]. Based on these observations GSTOs might be a significant modifier for arsenic-induced carcinogenesis. However, the magnitude of the association between GSTOs polymorphisms and As-induced cancers across different exposure levels has not been well-evaluated. Further studies with larger sample size, precise exposure assessment and large variation of exposure level are needed to draw these issues.

Both GSTO1 and GSTO2 are involved in arsenic methylation catalyzing the reduction of pentavalent arsenicals to trivalent arsenicals. Different GSTO polymorphisms may be of different capacity of arsenic metabolism, which may explain for variation susceptibility to arsenic. Inefficient methylation of arsenic has been reported to be associated with arsenic-induced skin lesions, skin cancers, bladder cancers and cardiovascular diseases [17-21]. Our previous study indicated that GSTO2 N142D GG genotype was associated with a higher percentage of iAs [31]. Two studies displayed that GSTO1 E155del was associated with markedly-changed percentage of iAs when compared to the wild homotype [32,50]. These studies suggest the effects of GSTOs polymorphisms on percentage of iAs. However, the association of GSTO1 with urinary arsenic metabolite pattern remains inconclusive because several studies did not show a significant association [28-30]. The inconsistent results among these studies might be due to very little concern for potential confounding factors such as ethnicity, nutritional status, As exposure level and other environmental factors. Better control of these confounding factors in further studies helps evaluation of real effect of GSTOs on arsenic methylation. In addition to MMA(V)/DMA(V) reductase activities, GSTOs also exhibits high thioltransferase activity as well as dehydroascorbate reductase activity and thus the enzymes could participate in intracellular thiol homeostatic reactions and antioxidant ascorbate recycling, respectively. Such enzyme activities reveal another possibility whereby changes in thioltransferase activity as well as ascorbate reductase activity may explain individual susceptibility to arsenic-induced health effects.

The present study had several limitations. Firstly, the small sample size with a limited number of cancer cases is a major limitation of this study. Based on such limited case numbers, a significant association of genetic markers with cancer risk is hard to reach after a Bonferroni correction for multiple comparisons. For this concern, we performed permutation tests to obtain the empirical *p*-value to overcome the size limitation and multiple testing issues (data not shown). Permutation is a non-parametric test and the empirical p-value can be obtained by calculating all possible test statistic under random rearrangements of the disease status on the study subjects [51]. The permutation tests performed for this study revealed the same results with that obtained by traditional statistical analysis, giving additional support for such gene-disease association. A second limitation is that about one-fourth of individual's CAE is missing, which decreases the power for the estimate of gene-environment interaction. Finally, the estimates of exposure status from questionnaires are subject to recall bias. However, the recall bias is considered to be non-differential between the cases and non-cases and thus the HRs should be underestimated. Notwithstanding these limitations, it is clear that this study was able to estimate the genetic effects of GSTT1 and GSTOs on the risk of UC, and, furthermore, the interactions between polymorphisms of such genes and high-level arsenic exposure can still be identified.

## Conclusion

We estimated the gene effects of members of GST superfamily on arsenic-induced urothelial carcinoma by long-term follow-up study in southwestern Taiwan. The results reveal the fact that the GSTs do not play a critical role in arsenic-induced urothelial carcinogenesis. However, the present data provide evidence that the effects of GSTT1 and GSTOs on arsenic-induced UC are possibly confined to high exposure level, where the subjects had UC risk almost twenty-fold higher than that of low exposure level. These observations are helpful for the identification of high risk group of urothelial carcinoma among arsenic-exposed people. In the future, the studies with a larger sample size, longer follow-up periods, markedly variation of exposure level as well as better control of confounding factors are needed to estimate single gene, gene-gene and gene-environmental interaction on the risk of adverse arsenic-induced health effects.

## List of abbreviations

As: arsenic; GST: glutathione S-transferase; GSTO: Glutathione S-transferase omega; UC: urothelial carcinoma; As(V): pentavalent arsenate; As(III): trivalent arsenite; MMA(V): monomethylarsonic acid; MMA(III): monomethylarsinic acid; DNA(V): dimethylarsinic acid; SAM: S-adeno methionine; AS3MT: arsenic (III) methyltransferase; SNPs: single nucleotide polymorphisms; BFD: blackfoot disease; CAE: cumulative arsenic exposure; PCR: polymerase chain reaction; RFLP: restriction fragment length polymorphism; HR: hazard ratio; CI: confidence interval; EF: etiologic fraction; ROS: reactive oxidative stress; w/w: wild type homozygous; w/v: heterozygous; v/v: variant homozygous

## Competing interests

We declare that we have no conflict of interest and none of the funding organization played a role in the design and conducted of this study; collection, management, analysis, and interpretation of the data; or preparation, review, and approval of the manuscript.

## Authors' contributions

CJC and LIH had full access to all of the data in the study and take responsibility for the integrity of the data and the accuracy of the data analysis. *Study concept and design: *CJC, LIH. *Acquisition of data: *CJC, LIH, WPC, YHC, WCL.

*Analysis and interpretation of data: *CJC, LIH. Drafting of the manuscript: LIH. *Critical revision of the manuscript for important intellectual content: *CJC.

*Experiment operating: *WPC, YHC, WCL. *Statistical analysis: *LIH, CJC.

*Obtained funding: *CJC. *Administrative, technical, or material support: *CJ Ch, LIH, TYY, YHW, YMH, HYC, MMW. *Study supervision: *CJC.

*All authors read and approved the final manuscript*.

## Supplementary Material

Additional file 1 **Sensitive analysis of the association of GSTs with the risk of urothelial carcinoma according to various cutoff points of arsenic exposure level**. Click here for file
