# Supplementary material for: Genetic polymorphisms in glutathione S-transferase (GST) superfamily and risk of arsenic-induced urothelial carcinoma in residents of southwestern Taiwan
Source: J Biomed Sci. 2011 Jul 29;18(1):51. doi: 10.1186/1423-0127-18-51 (PMC3199751; doi:10.1186/1423-0127-18-51)
Supplement: Additional file 1 — Sensitive analysis of the association of GSTs with the risk of urothelial carcinoma according to various cutoff points of arsenic exposure level. [file 1423-0127-18-51-S1.PDF]

Supplement table: Sensitive analysis of the association of GSTs with the risk of urothelial carcinoma according to various cutoff points of arsenic exposure level

	CAE<14.0	CAE≧14.0	CAE<17.5	CAE≧17.5	CAE<20	CAE≧20	CAE<21.2	CAE≧21.2
GSTM1								
Non-null	1.00	1.00	1.00	1.00	1.00	1.00	1.00	1.00
Null	0.93 (0.06-15.78)	0.81 (0.39-1.66)	0.67 (0.15-3.05)	0.88 (0.39-1.96)	0.69 (0.17-2.81)	0.90 (0.40-2.03)	0.60 (0.18-1.98)	1.03 (0.42-2.50)
GSTT1								
Non-null	1.00	1.00	1.00	1.0	1.00	1.00	1.00	1.00
Null	0.00	1.98 (0.90-4.35)	0.34 (0.06-1.83)	2.59 (1.03-6.51)*	0.27 (0.05-1.37)	3.25 (1.20-8.80)*	0.50 (0.14-1.74)	3.87 (1.28-11.70)**
GSTP1								
AA	1.00	1.00	1.00	1.00	1.00	1.00	1.00	1.00
AG	2.52 (0.16-40.69)	1.07 (0.44-.56)	0.48 (0.06-4.02)	1.29 (0.51-3.25)	0.41 (0.05-3.35)	1.41 (0.55-3.62)	1.22 (0.32-4.75)	0.99 (0.35-2.84)
GG	0.00	1.57 (0.51-4.88)	0.00	1.75 (0.54-5.72)	0.00	1.76 (0.54-5.73)	1.53 (0.17-13.33)	1.32 (0.35-4.91)
GSTO1-140								
CC	1.00	1.00	1.00	1.00	1.00	1.00	1.00	1.00
CA	0.00	0.55 (0.20-1.46)	0.41 (0.05-3.53)	0.63 (0.21-1.90)	0.32 (0.04-2.73)	0.70 (0.23-2.10)	0.26 (0.03-2.10)	0.77 (0.25-2.37)
AA	0.00	2.59 (0.59-11.42)	0.00	2.89 (0.65-12.90)	0.00	4.79 (1.03-22.39)*	0.00	4.68 (0.99-22.08)*
GSTO2 (-183)								
AA	1.00	1.00	1.00	1.00	1.00	1.00	1.00	1.00
AG	0.00	0.55 (0.23-1.32)	0.00	0.77 (0.31-1.91)	0.23 (0.03-2.02)	0.71 (0.28-1.84)	0.39 (0.08-1.90)	0.65 (0.23-1.83)
GG	0.00	2.13 (0.62-7.31)	2.07 (0.23-18.73)	1.89 (0.42-8.38)	2.04 (0.23-17.88)	2.90 (0.61-13.66)	1.43 (0.17-11.82)	3.69 (0.79-17.14)

GSTO2-142								
AA	1.00	1.00	1.00	1.00	1.00	1.00	1.00	1.00
AG	0.00	1.00 (0.46-2.14)	1.02 (0.23-4.59)	0.98 (0.41-2.32)	1.17 (0.28-4.79)	0.88 (0.37-2.14)	1.06 (0.32-3.54)	0.84 (0.33-2.18)
GG	0.00	2.19 (0.62-7.77)	0.00	2.25 (0.63-8.03)	0.00	2.53 (0.71-8.99)	0.00	3.58 (0.98-13.11)
GSTO diplotype								
CAA/CAA	1.00	1.00	1.00	1.00	1.00	1.00	1.00	1.00
CAA/AGG	0.00	0.57 (0.18-1.77)	0.00	0.76 (0.24-2.39)	0.00	0.79 (0.25-2.49)	0.00	0.88 (0.27-2.82)
AGG/AGG	0.00	3.07	0.00	2.99 (0.65-13.78)	0.00	4.91 (1.02-23.74)*	0.00	5.09 (1.03-25.15)*
Others	0.00	(0.67-14.01)	3.35 (0.64-17.51)	1.12 (0.39-3.20)	3.45	0.85 (0.27-2.65)	2.14 (0.57-8.02)	0.98 (0.31-3.14)
		1.53 (0.63-3.69)			(0.75-15.78)			

*p <0.05 ** p<0.01

GST: glutathione S-transferase; CAE: cumulative arsenic exposure
